# Antioxidant activity of ethanolic extract of inflorescence of *Ormenis Africana *in vitro and in cell cultures

**DOI:** 10.1186/1476-511X-10-78

**Published:** 2011-05-16

**Authors:** Riadh Ben Mansour, Bochra Gargouri, Mohamed Bouaziz, Nésrine Elloumi, Imtinène Belhadj Jilani, Zaineb Ghrabi, Saloua Lassoued

**Affiliations:** 1 Unité de recherche Biotechnologie et pathologies, Institut Supérieur de Biotechnologie de Sfax, Tunisia; 2 Laboratoire des Bioprocédés, Pôle d'Excellence Régionale AUF, (PER-LBP) Centre de Biotechnologie de Sfax, BP: «1177», 3038 Sfax, Tunisia; 3 Laboratoire de production fouragère et pastorale, Institut National d'Agronomie de Tunisie, Tunisia

## Abstract

**Background:**

The antioxidant potency of the hydroethanolic extract of *Ormenis Africana *(HEOA), Asteraceae was evaluated with regards to total polyphenol, flavonoid and anthocyanins content. Antioxidant activity has been assessed chemically and biologically. First, the free radical scavenging ability of HEOA was evaluated using two commonly in vitro tests: ABTS and DPPH radicals. Then, the protection effect of this extract against oxidative stress was conducted in HeLa cells treated with Fe^2+ ^or H_2_O_2. _Oxidative stress was evaluated by measuring the lipid peroxidation levels (TBARs and DC) and the antioxidant enzymes activities (catalase and Superoxide dismutase). Cytotoxic effect of HEOA was prealably determined against HeLa cell line by MTT assay.

**Results:**

HEOA contain considerable levels of antioxidant compound as evidenced by high amount of polyphenols (312.07 mg GAE/g dray matter), flavonoids (73.72 ± 1.98 mg QE/g dray matterl) and anthocyanins (0.28 ± 0.09 mg Cy-3-glu E/g dray matter). DPPH and ABTS assays showed a high antioxidant activity (IC_50 _= 24 μg/ml; TEAC = 2.137 mM) which was comparable to BHT.

In biological system, HEOA exhibited a 50% cytotoxic concentration evaluated as 16.52 μg/ml. Incubation of HeLa cell line with no cytotoxic concentrations resulted in a remarkable protection from oxidative stress induced by Fe^2+ ^or H_2_O_2 _which was evidenced by a decrease of MDA and CD levels as well as a diminution of antioxidant enzymes activities (Catalase and SOD) as compared to cells treated with Fe^2+ ^or H_2_O_2 _alone.

**Conclusion:**

The hydroethanolic extract of *O. Africana *could thus be considered as a source of potential antioxidants. The results of this study will promote the reasonable usage of this plant in food and pharmacy industries as well as in alternative medicine and natural therapy.

## Background

Plants have been used for medicinal purposes since time immemorial. They have been screened for their potential uses as alternative remedies for the maintenance of health. It has been estimated by WHO (World Health Organization) (2002) that about 90% of the world's population from developing countries rely mainly on traditional medicines (mostly derived from plants) for their primary health care [1,2]. Plant compounds are used for protection and treatment of human diseases such as coronary heart disease and cancer. They are also used as a food conservator. The therapeutic benefits of the medicinal plants are often attributed to their antioxidant properties in relation to their large content on antioxidant compounds such as vitamin C, Vitamin E, polyphenol and flavonoïdes [3-6].

*Ormenis Africana *has been used in several centuries for traditional medicine. This plant is endemic in North Africa (Tunisia, Algeria and Morocco). In Tunisia, it is traditionally used for its hypoglycemic effect as well as for the treatment of stomacal pain. Traditionally the inflorescences of this plant are mixed with honey and used for the treatment of the cardialgia ulcer and stomacal pain.

As far as we know, there is no scientific exploration of the antioxidant capacity of *O Africana *extract. In this regard, the purpose of this study was to evaluate the hydroethanolic extract of this plant as new potential source of natural antioxidants. First, the investigation of the flaavonoid, anthocyanin and total polyphenol content of the extract was conducted. The antioxidant activity was then chemically determined by in vitro assay (DPPH and ABTS scavaging activity) and biologically using the HeLa cell line. The cytotoxic effect of this extract was preliminary determined by MTT assay so all the used concentrations didn't show any cytotoxic affect for the cell culture.

## Results

### Total phenolic, anthocyanins and flavonoid compounds (chemical composition)

First, we was interested to evaluate the levels of anthocyanins, total phenolic and flavonoïd compounds in the hydroethanolic extract of *Ormenis Africana *(HEOA) using pH differential method, Follin-Ciocatleau colorimetric and AlCl_3 _methods, separately. The results show that HEOA contain 0.28 ± 0.09 mg Cy-3-glu E/ g DM, 312.07 ± 4,81 mg GAE/g DM and 73.72 ± 1.98 mg QE/g DM of the assessed compounds respectively (Table 1).

**Table 1:** Concentration of total phenolics, flavonoïds and anthocyane in hydroethanolic extract of *Ormenis Africana *(HEOA) inflorescences

Total phenol content (mg GAE/g dray matter)	312.07 ± 4,81
Flavonoides (mg QE/ g dray matter)	73.72 ± 1.98

Anthocyane (mg Cy-3-glu E/ g dray matter)	0.28 ± 0.09

### Antioxidant potential

The HEOA was screened for its antioxidant capacity by DPPH and ABTS radicals scavenging assays (Table 2). It exerted an antioxidant activity which was comparable to that BHT as shown by ABTS assay (TEAC = 2.137 vs 2.81 mM). However, the DPPH assays shown that BHT exhibited higher antioxidant activity than the plant extract (IC_50 _= 24 vs 8.31 μg/ml).

**Table 2:** ABTS and DPPH IC__50 __values for the hydroethanolic extract of *Ormenis Africana *(HEOA) flowering summits and BHT

Sample	ABTS values (TEAC)	**DPPH IC**_**50 **_**values (μg/ml)**
HEOA	2.137 ± 0.12	24 ± 1.57

BHT	2.81 ± 0,13	8.31 ± 0.2

### Cytotoxicity effect of *Ormenis Africana *extract

**Figure 1:**
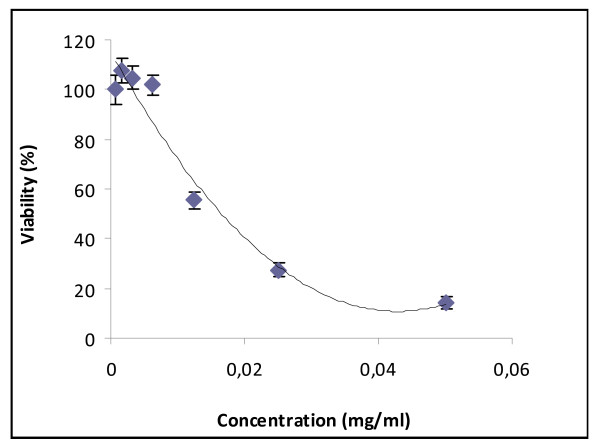
**Cytotoxic effect of HEOA on HeLa cell line. **The inhibitory effect of different doses on cell growth was determined by MTT assay. Cells were treated with HEOA at concentration ranging from 0 to 50 μg/ml. the percent growth reduction was calculated from the extinction difference between treated cell culture and the control. Results are the means of three repetitions

To investigate the cytotoxic effect of HEOA on HeLa human cell line, cells were treated with various concentrations of HEAO ranging from 0 to 50 μg/ml for 72 hours, and then submitted to the MTT test (figure 1). Data showed that HEOA displayed an inhibition effects on human cells growth in a dose dependent manner. The IC_50 _of HEOA was evaluated to 16.52 μg/ml. Hence doses under this concentration were used for biological antioxidant activity investigation. Two doses of the extract were chosen: 5 and 10 μg/ml which induce less than 20% cytotoxicity.

### Biological antioxidant activity in human cell culture

#### Lipid peroxidation

The investigation of the biological antioxidant activity of HEOA was carried in the HeLa human cell line. Cells were cultured with or without addition of HEOA for 72 hours. Oxidative stress was induced by adding 100 μM Fe^2+ ^solution (as Fe_2_SO_4_) in PBS for one hour. Malondialdehyde and conjugated diene production, two lipid peroxydation markers were evaluated.

**Figure 2:**
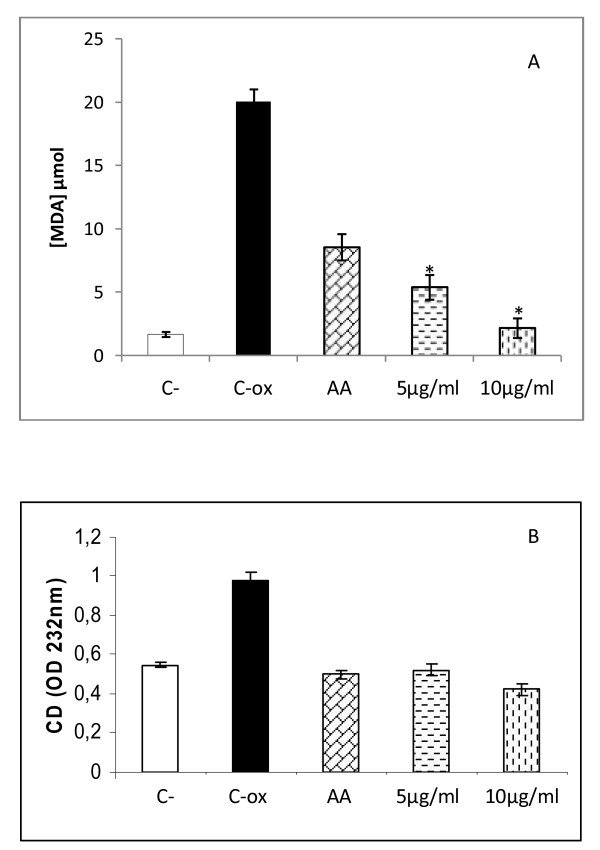
**MDA (A) and conjugated diene (B) levels in HEOA supplemented HeLa cell line**. Cells were cultured in 25 cm^2 ^flasks with 5 and 10 μg/ml of HEOA for 72 hours. Oxidative stress was induced by addition of Fe2+ to the cells for 1 hour at a final concentration of 100 μM. TBARs and conjugated diene (CD) were compared to untreated cells (C-), cells treated with Fe2+ alone (C-ox) and cells treated with 100 μM ascorbic acid (AA).

The oxidative treatment resulted in at least 13 fold increase in TBARs concentration compared with control cells. As shown in figure 2a, a significant protection against ROS inducing damage was obtained with both used concentrations in a dose dependent manner. In fact, a significant decrease in TBARs level was obtained with the concentration of 5 μg/ml as compared to Fe^2+ ^treated cells (p < 0.05), while a total reestablishment of TBARs level was obtained with the concentration of 10 μg/ml as compared to untreated cells (p > 0.05).

Concomitantly, HeLa cells treatment with HEOA exhibited an antioxidant effect evidenced by a decrease in conjugated diene level with both used concentrations as compared with Fe^2+ ^treated cells alone. In contrast to TBARs data, there is no correlation between the concentrations used and the antioxidant activity was observed (figure 2b).

#### Antioxidant enzyme activities

Bioactivity of HEOA on SOD and catalase antioxidant enzymes was measured in HeLa cells. As shown in table 3, induction of oxidative stress with H_2_O_2 _led to an increase in the SOD and catalase activities which can be explained by an adaptation of the enzymatic antioxidant system of the cells to the ROS production. Interestingly, the treatment of cells with both concentrations of HEOA induced a significant decrease in the catalase activities (p < 0.05). A slight decrease in SOD activity was also obtained after treatment with HEOA but it was not statistically significant (p > 0.05).

**Table 3:** Effect of HEOA cells pretreatment on Catalase and Superoxide dismutase activities

Enzymes	Catalase (U/ml)	SOD (% of inhibition)
C-	44.5 ± 7.77	26.5 ± 4.94

C-ox	126.5 ± 9,19	90 ± 7.07

HEOA (μg/ml)		

5	58.16**±5,8	75 ± 7.9

10	43.5**±4,9	69 ± 6.7

## Discussion

Plants contain different groups of phenolic compounds, including simple phenolics, phenolic acids, anthocyanins, hydroxycinnamic acid derivatives and flavonoids. All the phenolic classes have received considerable attention because of their physiological functions, including free radical scavenging [7]. The antioxidant activity of phenolics is mainly due to their redox properties which make them act as reducing agents, hydrogen donors, and singlet oxygen quenchers. They may have also a metal chelating potential [8].

In this study, we assessed for the first time, the chemical composition of the HEOA of *Ormenis Africana *specie. The content on total polyphenols, anthocyanins and flavonoids was determined. No references concerning the total phenolic content of OA species could be found despite the thorough literature survey. These results showed that the extract is rich on total polyphenol content as compared to plants that belong to the same family (Asteraceae) such as the Helichrysum noeanum (312.07 vs 163.63 mg GAE/g DM) [9-11]. Besides, flavonoids and anthocyanins, as estimated by pH differential and AlCl_3 _methods respectively are present in high concentrations compared to other alcoholic extracts from other Asteraceae species [10]. These preliminary results, suggest an anti-radical property of the HEOA. The free radical scavenging activity was determined through the DPPH test and the ABTS assay. The concentration of antioxidant needed to decrease the initial DPPH concentration by 50% (IC_50_) is a parameter widely used to measure antioxidant activity. As the IC_50 _value of the extract decreases, the free radical scavenging activity increases. The investigated extract expressed the ability to scavenge the stable DPPH free radical reaching 50% of reduction with an IC_50 _values of 24 μg/ml which is higher than the IC_50 _determined by the BHT as a positive control (8,31 μg/ml). However, the same extract showed a comparable antioxidant activity to BHT using the ABTS radical. Indeed, the IC_50 _of our extract was evaluated at 2.1 mM TEAC versus 2,81 for the BHT. Indeed, in the present study it is found that the aqueous ethanol extract of *Ormenis Africana *inflorescences contains substantial amount of phenolics and it is the extent of phenolics present in this extract being responsible for its marked antioxidant activity as assayed through tow in vitro models. Several reports have shown close relationship between total phenolic contents and antioxidative activity of the fruits, plants and vegetables [11-14]. The chemical composition and chemical structures of active extract components are important factors governing the efficacy of natural antioxidants. Concerning the HEOA, further studies are needed for the isolation and identification of individual phenolic compounds and the assessment of their antioxidant activities. For instance, it has already been reported that phenolic compounds with ortho- and para-dihydroxylations or a hydroxy and a methoxy group or both are more effective than simple phenolics [15].

Our results are in agreement with those described for other extracts from plants belonging to the *Asteraceae *family. Indeed, the methanolic extracts and fractions from Achillea showed scavenging activity towards hydroxyl radical in different *in vitro *systems [16,17]. The ethyl acetate and the butanol fraction of Achillea extract showed a significant reduction of hydroxyl radical in different in vitro systems such as liver homogenate, hemolyzed blood, serum and postmitochondrial liver fraction [17]. The hydroethanolic extract of *Ormenis Africana *exhibited a higher antioxidant scavenging activity compared to other alcoholic extracts from Helichrysum and Achillea (methanol or ethanol) [11,18].

The biological antioxidant activity of HEOA was also investigated in the HeLa cell culture. Considering that the chemical composition of the HEOA is not determined previously, we initially carried out control experiments to assess the cytotoxicity of our extract on HeLa cell line using MTT assay. The results showed that the extract had a cytotoxic effect in a dose dependent manner with an IC_50 _value evaluated as 16,52 μg/ml. In order to investigate the antioxidant activity of our extract on cells model, we choose two concentrations that induce less than 20% of toxicity according to the results showed in figure 1. The concentrations used are 5 and 10 μg/ml in all the experiences. HeLa cells are subjected to oxidation by Fe^2+ ^solution (Fe_2_SO_4_), to assess lipid peroxidation, or hydrogen peroxide, to assess antioxidant enzymes activities. The oxidative treatment with 100 μM Fe^2+ ^resulted in the increase of MDA and CD levels due to the enhancement of the lipid peroxidation reaction. The pretreatment of cells by the HEOA resulted in the reduction of the production rate of the two considered markers as shown in figure 2. Moreover, the addition of H_2_O_2 _in the culture medium resulted in an increase of the catalase and superoxide dismutase activities. It's well known that catalase convert hydrogen peroxide into oxygen and water. The increase of the activity of this enzyme can be considered as an adaptation response to the addition of H_2_O_2 _in the culture media. The standard substrate of SOD is the superoxide anion which was dismutated to hydrogen peroxide. However, it was recently reported that at high level of H_2_O_2 _this enzyme, especially the MnSOD, enhanced the reverse reaction yielding to superoxide anion [19] which can explain the increased SOD activity after the treatment with hydrogen peroxide. The addition of the HEOA in the culture medium restored the activity of catalase (table 3) and lead to a decrease in SOD activity. This result could be explained by the reestablishment of the oxidant/antioxidant balance in the cell line and confirm the antioxidant property of our extract. This antioxidant activity could be explained by the high content of the HEOA on polyphenol, flavonoid and anthocyanins. Our results are in agreement with those found by other reports. In fact, Tuberoso et al (2009) [20] reported that the hydroalcoholic extracts from Achillea spp (Asteraceae) reduce significantly the production of MDA on caco-2 pretreated cells.

The results of this study revealed that the hydroethanolic extract of *Ormenis Africana's *inflorescences contain a considerable amount of polyphenol, flavonoid and anthocyanin compounds, and had significant antioxidant activity as determined by chemical and biological assays. The inflorescence of *Ormenis Africana *could be used as a potential source of natural antioxidants and bioactive molecules.

## Materials and methods

### Plant materials and extraction procedure

The inflorescence of *Ormenis africana *was harvested from the region of Kef (North West of Tunisia) and used as plant materials in this work. The inflorescences were dried at room temperature and 100 g of plant material were treated overnight with water:ethanol 20:80 (v/v) under gentile stirring. The hydroethanolic extract was filtered through a cellulose filter, lyophilized and frozen at -80°C until use.

### Total phenol determination

Total phenols were determined by using the Folin-Ciocalteu reagent according to the method of Singleton and Rossi [21]. Briefly, a 50 μl aliquot of the extract was assayed with 250 μl Folin reagent and 500 μl of sodium carbonate (20%, w/v). The mixture was vortexed and diluted with water to a final volume of 5 ml. After incubation for 30 min at room temperature, the absorbance was read at 765 nm. Total phenols were expressed as gallic acid equivalents (GAE), using a calibration curve of a freshly prepared gallic acid solution. For the gallic acid, the curve absorbance versus concentration is described by the equation *y *= 0.0012*x*- 0.0345 (*R*^2 ^= 0.9997).

### Total flavonoid Determination

Total flavonoids were measured by a colorimetric assay developed by Zhishen et al (1999) [22]. One millilitre of a diluted sample was added to 4 mL of water and 300 μl of NaNO_2 _(5%, v/v) in water was added. After 5 min of incubation, 300 μL of 10% AlCl_3 _was added. After 6 min, 2 mL of aqueous NaOH (1M) was added to the mixture. Immediately; the mixture was diluted with water to 10 mL. The absorbance was read at 510 nm. Total flavonoids were expressed on a dry weight basis as quercetin equivalents, using a calibration curve of a freshly prepared quercetin solution. For quercetin, the curve absorbance versus concentration is described by the equation y = 0.0049x (R^2 ^= 0.9984).

### Total anthocyanins measurement using pH differential method

Total anthocyanins were measured according to a modification of the methods described earlier [23,24]. Two dilutions of the sample were prepared, one for pH 1.0 using potassium chloride buffer (0.03 M, 1.9 g KCl into 980 mL distilled water) and the other for pH 4.5 using sodium acetate buffer (0.4 M, 54.4 g CH_3_CO_2_Na. 3H_2_O in 960 mL distilled water). Samples were diluted 10 times to a final volume of 2 mL. The absorbance of each sample was measured at 520 nm against distilled water as blank. The samples had no haze or sediment and thus correction at 700 nm was omitted. The concentration (mg/L) of each anthocyanin was calculated according to the following formula:

and expressed as Cy-3-glc equivalents:

where *A *is the absorbance = (*A*_λvis-max_)_pH 1.0 _- (*A*_λvis-max_)_pH 4.5_, *MW *is the molecular weight (g/mol) = 449.2 g/mol for Cy-3-glc, *DF *is the dilution factor (0.2 ml sample is diluted to 2 ml, *DF *= 10), and e is the extinction coefficient (L x cm^-1 ^x mol^-1^) = 26,900 for Cy-3-glc, where *L *(path length in cm) = 1. For comparison, the same extinction coefficient was used for other standards to calculate the concentration of each anthocyanin and thus results reported is expressed as Cy-3-glc equivalents.

### Free radical scavenging activity

#### *DPPH Radical Scavenging Assay*

The hydrogen atom or electron donation ability of the corresponding extracts and some pure compounds was measured from the bleaching of purple coloured methanol solution of DPPH. The DPPH (1,1-diphenyl-2-picrylhydrazyl) radical-scavenging effect was evaluated following the procedure described in a previous study [25]. In succinct terms, aliquots (50 μL) of various concentrations of the test compound in methanol were added to 5 mL of a 0.004% methanol solution of DPPH. After a 30 min incubation period at room temperature the absorbance was read against a blank at 517 nm. Inhibition free radical DPPH in percent (I%) was calculated in the following way: I% = [(A_blank _- A_sample_)/A_blank_] x 100, where A_blank _is the absorbance of the control reaction (containing all reagents except the test compound), and A_sample _is the absorbance of the test compound. Test compound concentration providing 50% inhibition (IC_50_, expressed in μg mL^-1^) was calculated from the graph plotted inhibition percentage against extract concentration. Synthetic antioxidant butylated hyroxytoluene (BHT) was used as positive control and all tests were carried out in triplicate.

#### *Trolox equivalent antioxidant capacity (TEAC)*

The Trolox equivalent antioxidant capacity (TEAC) measures the reduction of the radical cation of ABTS by antioxidants. This assay was performed as previously described [26]. Briefly, ABTS radical cation (ABTS^•+^) was produced by reacting ABTS stock solution with 2.45 mM potassium persulfate (final concentration) and by allowing the mixture to stand in the dark at room temperature for 12- 24 h before use. For the study of phenolic compounds the ABTS^•+^, the solution was diluted with water to an absorbance of 0.70 (±0.02) at 734 nm. For the photometric assay 1 ml of the ABTS^•+^, the solution and 100 μL antioxidant solution were mixed for 45 s and measured immediately after 5 min at 734 nm (absorbance did not change significantly up to 10 min). Compounds were assayed at five different concentrations determined within the linear range of the dose-response curve. A calibration curve was prepared with different concentrations of Trolox (0-20 μM). Results were expressed in mM of Trolox.

### HeLa cell culture

The continuous human cell lines HeLa (epithelial cervical cancer cell line) was investigated for cytotoxicity and antioxidant effect of plant extracts. This adherent cell line was grown in RPMI 1640 medium (Gibco) supplemented with 10% (v/v) foetal calf serum (FCS) and 2 mM L-glutamin in tissue culture flasks (Nunc). It was passed twice a week and kept at 37°C in a humidified atmosphere of 95% air and 5% CO_2_.

### Induction of oxidative stress

Cells were adjusted to 5 10^5 ^cells/ ml in 25 cm^2 ^flasks, and incubated at 37 °C. Oxidative stress was induced, after 72 h, by addition of Fe^2+ ^(as Fe_2_SO_4_) to the cells at a final concentration of 100 μM, for 1 h. The oxidation was performed in phosphate buffered saline (PBS).

To evaluate superoxide dismutase (SOD) and catalase activities, oxidative stress was induced using 100 mM H_2_O_2 _during 1 hour. The activities of catalase and SOD were assessed in cell lysates.

### Malondialdehyde (MDA) determination

For evaluation of MDA production rate, thiobarbituric acid-reactive species (TBARs) assay was used. Adherent cells were detached using trypsin/EDTA solution and centrifuged at 3000 rpm for 10 min. The pellet was resuspended in 500 μL of deionized water and lysed by five cycles of sonication during 20 s at 35% (Sonisc, vibracell). One millilitre of TBA solution (15% trichloroacetic acid, 0.8% thiobarbituric acid, 0.25 N HCl) was added. The mixture was heated at 95°C for 15 min to form MDA-TBA adduct. Optical density (OD) was measured by a spectrophotometer (Biochrom, Libra S32) at 532 nm. Values were reported to a calibration curve of 1,1,3,3-tetraethoxypropane (1.1.3.3 TEP).

### Antioxidant effect

To assay the capacity of plant extract to protect HeLa cells from ROS-mediated oxidative injury, cells were preincubated for 72 h in the presence of different concentrations of ethanol extracts. At the end of the preincubation time, the medium was changed before the addition of the oxidative stress-inducing agent (100 μM of Fe_2_SO_4 _or 100 mM of H_2_O_2_). Finally, the above mentioned markers were evaluated.

Different controls were used: (i) HeLa cells without any treatment; (ii) HeLa cells with 100 μM Fe^2+^; (iii) HeLa cells with 100 μM of ascorbic acid

### MTT cell proliferation assay

The MTT (3-(4,5-dimethylthiazolyl-2)-2,5-diphenyltetrazolium bromide) cell proliferation assay measures the cell proliferation rate and conversely, the reduction in cell viability when metabolic events lead to apoptosis or necrosis. The yellow compound MTT (Sigma) is reduced by mitochondrial dehydrogenases to the water insoluble blue formazan compound, depending on the viability of the cells.

Cells (3 10^4 ^cells/ml) were grown on microtiter plates (200 μl of cell suspension/well) in 96 well microplates with serial dilutions of extract. 72 h later, 20 μl of a MTT solution (5 mg/ml in PBS) were added in each well. The plate was incubated for 4 h at 37°C in a CO_2 _incubator. After incubation, 180 μl of medium were removed from each well and 180 μl of DMSO/methanol (50:50) were added to each sample. The preparations were mixed thoroughly on a plate shaker with the cells containing formazan crystals. When all the crystals were dissolved, absorbance was measured at 570 nm with a microplate reader (Elx 800 microplate reader).

### Determination of catalase activity

Catalase activity was measured as described previously by Aebi [27]. This method is based on the principle that the absorbance at 240 nm decreases because of dismutation of H_2_O_2_. The amount of H_2_O_2 _converted into H_2_O and O_2 _in 1 min under standard conditions is accepted as the enzyme reaction velocity. The number of catalase units was determined as follows: U/mL = [(3.45*slope)/0.05]*(1000/50 μl).

### Determination of SOD activity

The SOD activity was determined by spectrophotometry (420 nm) using the pyrogallol assay as described previously [28] and modified as follows: the rate of autoxidation of pyrogallol in Tris-cacodylic acid diethylenetriaminepentaacetic acid (DTPA) buffer (pH 8-8.2) was determined (A1). The autoxidation of pyrogallol was evaluated under the same conditions after addition of 25 μl of cells lysate (A2). The percentage inhibition of pyrogallol oxidation was determined using the formula: % Inhibition = [(A1-A2)/A1]*100.

### Statistical analysis

Each value is the mean of three replications. Values of different parameters were expressed as the mean ± standard deviation (x ± SD). The one-way analysis of variance (ANOVA) was performed at the level of p < 0.05 to evaluate the significance of differences between mean values. Statistical analysis was performed using SPSS (SPSS 13 for Windows) statistical software.

## Abbreviations

DM: Dry matter; DPPH: 1,1-diphenyl-2-picrylhydrazyl; FCS: Foetal calf serum; GAE: Gallic acid equivalent; HEOA: Hydroethanolic extract of *Ormenis Africana; *MDA: Malodialdehyde; OA: *Ormenis Africana; *PBS: Phosphate buffer saline; ROS: Reactive oxygen species; SOD: Superoxide dismutase; TBA: Thiobarbuturic Acid; TBARs: Thiobarbuturic Acid Reactive species; TEAC: Trolox equivalent antioxidant capacity; TEP: 1,1,3,3-tetraethoxypropane.

## Competing interests

The authors declare that they have no competing interests.

## Authors' contributions

RBM and BG prepared the study design, carried out all the biological studies, analyzed and discussion of the data, and drafted the manuscript. MB helped with chemical analysis of the extract and correction of the manuscript. NE carried out some biological assays and helped with the manuscript preparation. IBJ and ZG give us the plant material. SL participated in the study design, discussion the data and helped to draft and correction of the manuscript. All authors have read and approved the final manuscript.
